# High Frequency Targeted Mutagenesis Using Engineered Endonucleases and DNA-End Processing Enzymes

**DOI:** 10.1371/journal.pone.0053217

**Published:** 2013-01-24

**Authors:** Fabien Delacôte, Christophe Perez, Valérie Guyot, Marianne Duhamel, Christelle Rochon, Nathalie Ollivier, Rachel Macmaster, George H. Silva, Frédéric Pâques, Fayza Daboussi, Philippe Duchateau

**Affiliations:** 1 Cellectis SA, Paris, France; 2 Cellectis Stem Cells, Genopole, Evry, France; University Medical Center Hamburg-Eppendorf, Germany

## Abstract

Targeting DNA double-strand breaks is a powerful strategy for gene inactivation applications. Without the use of a repair plasmid, targeted mutagenesis can be achieved through Non-Homologous End joining (NHEJ) pathways. However, many of the DNA breaks produced by engineered nucleases may be subject to precise re-ligation without loss of genetic information and thus are likely to be unproductive. In this study, we combined engineered endonucleases and DNA-end processing enzymes to increase the efficiency of targeted mutagenesis, providing a robust and efficient method to (i) greatly improve targeted mutagenesis frequency up to 30-fold, and; (ii) control the nature of mutagenic events using meganucleases in conjunction with DNA-end processing enzymes in human primary cells.

## Introduction

Engineered endonucleases such as meganucleases, zinc finger nucleases, and the recent transcription activator-like effector nucleases (TALEN) have revolutionized the post genomic area. By targeting cleavage to specific DNA sequences, such endonucleases can stimulate either homologous recombination (HR) or non-homologous end-joining (NHEJ) at predefined locations, making precise genome modifications possible. Whereas HR is used to insert a specific sequence at or nearby the break site, NHEJ, active throughout the cell cycle, is mainly exploited for gene inactivation purposes. Although genome modification studies have reported high frequencies of NHEJ events [1]–[12], perfect re-ligation of the broken DNA ends without loss of genetic information is probably the most frequent outcome.

Recent studies have unraveled the existence of two distinct NHEJ pathways [13], [14], [15]: the canonical DNA-PK dependent pathway (D-NHEJ), which requires a KU/DNA-PKcs/Lig4/XRCC4 complex, and an alternative NHEJ pathway (B or alt-NHEJ) that is employed in the absence of the former. While D-NHEJ, considered the predominant double-strand break (DSB) repair pathway, leads mainly to precise repair of the DNA DSB by ligating ends back together, the alt-NHEJ pathway is highly mutagenic. In contrast to classical NHEJ, HR and alt-NHEJ pathways share the same initiation event of ssDNA resection. Controlling the initiation event is therefore essential to the final outcome of the DSB repair and thus for maintaining genome integrity [13].

Homing endonucleases (HE), also known as meganucleases, recognize long DNA targets (14–40 bp). In nature, HEs are usually coded within mobile introns or inteins and could be considered as genomic parasites since they promote the propagation, via a mechanism of DSB-induced homologous recombination, of their own ORF into the homologous allele lacking the mobile element. Recent advances in protein engineering have made it possible to successfully redesign the protein-DNA interface of several HEs in order to change their specificity [16]–[29], making virtually every gene within reach of genome engineering techniques. Among available strategies, targeted mutagenesis by a NHEJ mechanism represents an attractive approach for gene inactivation as there is no need for a repair plasmid and efficacy is likely less cell-type dependent since NHEJ appears to be active throughout the cell cycle. However, many of the DNA breaks produced by engineered HEs are subject to faithful repair and thus strategies to control the DSB-induced pathway are of interest.

In this study, we provide a robust and efficient method to (i) greatly improve targeted mutagenesis frequency up to 30-fold, and; (ii) control the nature of mutagenic events in human primary cells using meganucleases in conjunction with DNA-end processing enzymes.

## Materials and Methods

### Nucleases

Nucleases quoted in this study are listed in Data S1.

### Culture condition

Human 293H cells (Life Technologies, Carlsbad, CA) and hamster CHO-K1 cells (ATCC) were cultured at 37°C with 5% CO2 in complete medium DMEM and F12-K, respectively, supplemented with 2 mM L-glutamine, penicillin (100 U/ml), streptomycin (100 µg/ml), amphotericin B (Fongizone: 0.25 µg/ml, Life Technologies,) and 10% FBS. The human primary fibroblasts Detroit 551 (ATCC), derived from fetal skin, were cultured in MEM supplemented with 15% FBS, 1% GlutaMAX™ and 1% penicillin-streptomycin. iPS cells used for this study were provided by the Cardiovascular Research Center, Mount Sinai School of Medicine, New York, NY 10029 [30]. They were cultured on mouse embryonic fibroblasts (MEF)-feeder layers in human stem cells medium: DMEM/F12 (Life Technologies Corporation, USA), supplemented with 25% knock-out serum replacement (Life Technologies Corporation, USA), 50 µM 2-mercaptoethanol (Life Technologies Corporation, USA), 1X Non Essential Amino Acids (Life Technologies Corporation, USA) and 10 ng/mL bFGF2 (Life Technologies Corporation, USA). MEF-conditioned medium is obtained by culture of MEF feeder with stem cell medium during 24 h.

### Extrachromosomal activity and survival assays in CHO-K1

Activity and toxicity in mammalian cells was measured as previously reported by Grizot *et al*. [31] For extrachromosomal assays, CHO-K1 cells were seeded in 96-well plates at 2,500 cells per well and transfected one day post platting with 150 or 200 ng of total DNA using Polyfect transfection reagent according to the supplier's protocol (Qiagen). In the survival assay, 10 ng of GFP-encoding plasmid was mixed with various amounts (from 2.5 to 25 ng) of meganuclease expression vectors.

### NHEJ cellular model

The construct monitoring NHEJ is made of an ATG start codon followed by (i) the HA tag sequence; (ii) a GS meganuclease (GSm) recognition site; (iii) a glycine-serine stretch, and; (iv) a GFP reporter gene lacking the start codon. In this arrangement the GFP gene is inactive due to a frame-shift introduced by the GS recognition site, however, creation of a DNA DSB by GSm, followed by a mutagenic NHEJ repair event, can lead to restoration of GFP gene expression in frame with the ATG start codon.

This sequence was cloned into the human RAG1 endogenous locus using the plasmid hsRAG1 Integration Matrix CMV Neo from cGPS® Custom Human Full Kit DD (Cellectis Bioresearch). The plasmid contains all the necessary components to obtain by homologous recombination a highly efficient insertion event of the transgene at the RAG1 endogenous locus. It is composed of two homology arms of 1.8 and 1.2 kbp separated by an expression cassette of the neomycin resistance gene driven by a mammalian CMV promoter and our transgene under a second CMV promoter. It also contains the HSV-TK gene under EF1α promoter control placed outside the homology arms. This plasmid was transfected in 293H cells and clones presenting double resistance (neomycin and ganciclovir) were used to quantify NHEJ induced by GSm.

### Transfection in the NHEJ cellular model

One million cells were seeded one day prior to transfection. These cells were co-transfected with 3 µg of plasmid encoding GSm or scTrex-GSm, with or without 2 µg of plasmid encoding Tdt, Trex or scTrex in 5 µg of total DNA by complementation with a pUC vector using 25 µl of lipofectamine (Invitrogen) according to the manufacturer's instructions. Four days post- transfection, cells were harvested and the percentage of GFP-positive cells was measured by flow cytometry analysis using Guava instrumentation (Millipore). Genomic DNA was extracted from cell population and locus specific PCRs were performed using the following primers: 5′-CCATCTCATCCCTGCGTGTCTCCGACTCAG (forward adaptor sequence)-10N (sequences needed for PCR product identification)-GCTCTCTGGCTAACTAGAGAACCC (transgenic GS locus specific forward sequence)-3′ and 5′-CCTATCCCCTGTGTGCCTTGGCAGTCTCAG-(reverse adaptor sequence)-TCGATCAGCACGGGCACGATGCC (transgenic GS locus specific reverse sequence). PCR products were sequenced by a 454 sequencing system (454 Roche). Several thousand sequences were obtained per PCR product and then analyzed for the presence of site-specific insertion or deletion events at the GS cleavage site (Table S2). The analysis did not consider single-base insertion or deletion events in order to avoid sequencing mistakes being defined as a mutation events.

### Transfection in 293H cells to monitor meganuclease-induced mutagenesis at endogenous loci

293H cells were plated at a density of 1×10^6^ cells per 10 cm dish. The next day, 3 µg of plasmid encoding the meganucleases RAG1m, DMD21m or CAPNS1m, respectively, were co-transfected with or without 2 µg of plasmid encoding Tdt in 5 µg total DNA by complementation with a pUC vector. Cells were harvested 7 days post-transfection for genomic DNA extraction and locus specific PCR for amplicon sequencing analysis. The same amount of meganuclease was co-transfected with or without 5 µg of Trex or scTrex encoding plasmids in 10 µg of total DNA. Cells were harvested 3 days post-transfection for genomic DNA extraction and locus specific PCR for amplicon sequencing analysis. Locus specific PCRs were performed using the following primers containing the adaptor sequences needed for amplicon sequencing (see above) and sequence specific to the loci: for CAPNS1 For_C: 5′-CGAGTCAGGGCGGGATTAAG-3′ and Rev_C: 5′-CGAGACTTCACGGTTTCGCC-3′; for RAG1 For_R:5′-GGCAAAGATGAATCAAAGATTCTGTCC-3′ and Rev_R:5′-GATCTCACCCGGAACAGCTTAAATTTC-3′ for DMD21 For_D: 5′GCTGTTATCTCAGTCACAAATACACATCTG-3′ and Rev_D:5′ CCTCTTTGCAACAATTCTTTTACAGTACCC-3′. Several thousand sequences were obtained per PCR product and then analyzed for site-specific insertion or deletion events (Table S2).

### Transfection in Detroit 551 cells to monitor meganuclease-induced mutagenesis at CAPNS1 locus

Electroporation was carried out with the NHDF nucleofactor kit and device (Lonza group Ltd, Switzerland) under the U-020 transfection program. Cells (1×10^6^) were transfected with 6 µg of CAPNS1 meganuclease coding plasmid (fused or not to scTrex endonuclease) and 4 µg of Tdt, scTrex or pUC. A total of 10 µg of DNA was used per transfection reaction. Cells were then plated in 6-well plates and cultivated during 72 h before to be collected for genomic DNA extraction and amplicon sequencing analysis.

### Transfection in iPS cells to monitor meganuclease-induced mutagenesis at CAPNS1 locus

Two days before transfection, cells were treated with CDK dissociation solution (ReproCELL Incorporated, Japan), transferred on Geltrex (Life Technologies Corporation, USA) coated dishes and cultivated with MEF-conditioned stem cell medium. The day of transfection, cells were pre-incubated one hour with 10 µM Y-27632 Rock inhibitor. Electroporation was carried out with the Human Stem cells nucleofector solution 2 (Lonza group Ltd, Switzerland) using B-016 transfection program. Cells (1×10^6^) were transfected with 6 µg of CAPNS1 meganuclease coding plasmid (fused or not to scTrex endonuclease). A total of 10 µg of DNA was used per transfection reaction. Cells were then seeded on Geltrex pre-coated 6-well plates and cultivated during 72 h in MEF-conditioned stem cell medium (changed daily) before being collected for genomic DNA extraction and amplicon sequencing analysis.

### Creating single-chain TREX2 (scTrex)

A linker of 11 amino acids (TPPQTGLDVPY) was designed to bridge the C-terminal alanine of the N-terminal Trex2 molecule to the serine at the N-terminus of the second Trex2 molecule in the homodimer. To create the single-chain molecule, a strategy was adopted using a unique PstI restriction site within the Trex DNA sequence. Briefly, the Trex2 coding sequence was cloned into a mammalian expression vector (pcDNA3.1) and primers were designed to cover the PstI site (PstI_for/PstI_rev), along with primers encompassing either a region of the N-terminal Trex sequence and the linker (Trex2Link_for); or part of the C-terminal Trex2 sequence plus the linker (Trex2Link_rev). Two independent PCR's were carried out creating two products for use as template in an assembly PCR using the PstI_for and PstI_rev primers. The resulting product was digested by PstI and ligated into the vector containing Trex2, also digested by the same enzyme, creating the single-chain Trex2 with the 11 amino acid linker.

### Fusing scTrex to a meganuclease

To create scTrex-meganuclease fusions, we first fused Trex2 to a meganuclease at its N-terminus, using a ten amino acid glycine-serine stretch (GGGGS)_2_ as a linker. Fusion protein constructs were obtained by separately amplifying the two ORFs using primer pairs Link10MNRev and CMV_for (5′-CGCAAATGGGCGGTAGGCGT-3′) and Link10MNFor and V5reverse (5′-CGTAGAATCGAGACCGAGGAGAGG-3′), which are located on the plasmid backbone. A PCR assembly was carried out using the CMVfor/V5reverse oligonucleotides. As for both Trex2 and the meganuclease, the final PCR product was then digested by AscI and XhoI and ligated into the pcDNA3.1, also digested with these same enzymes. To create the scTrex fusion variants, each Trex-meganuclease fusion was cut at a unique Tth111I restriction site, followed by insertion of the fragment excised from a similarly digested scTrex plasmid, leading the final scTrex2-megnuclease molecule.

### Statistical analysis

Error bars represent SEM. *p* values are calculated using the Student's two-tailed paired t-test between samples indicated. * represents *p*<0.05, ** represents *p*<0.005, and *** represents *p*<0.0005.

## Results and Discussion

**Figure 1:**
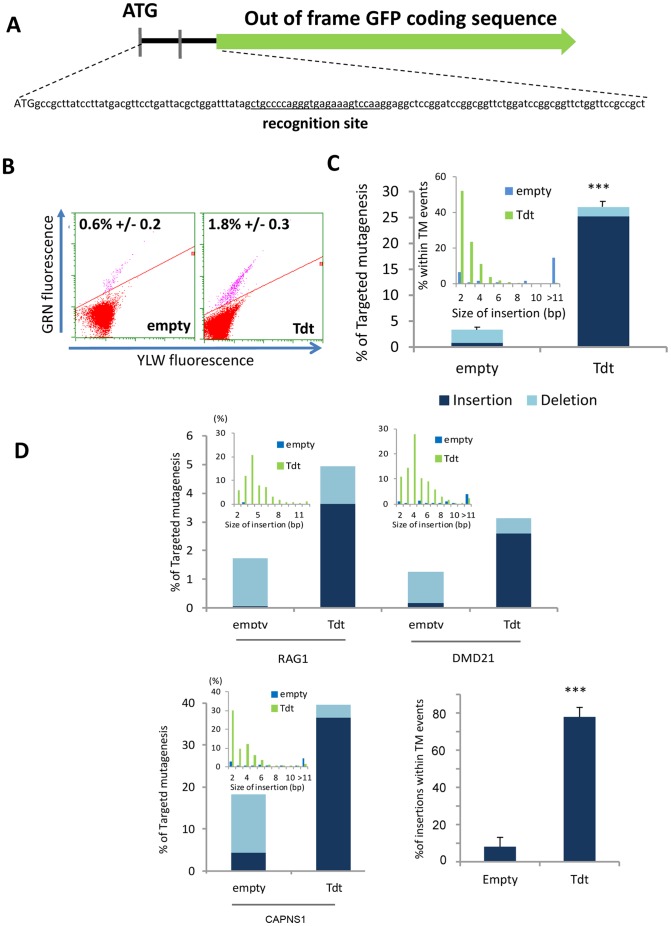
Effect of Tdt on meganuclease-induced mutagenesis. (A) Schematic representation of the transgene measuring NHEJ activity. A GFP gene lacking the ATG start codon was cloned out of frame and downstream of an exogenous sequence containing a meganuclease recognition site. (B) Quantification by flow cytometry of GFP positive cells 3 days post transfection with meganuclease alone (empty) or with meganuclease and Tdt (Tdt); experiments performed in triplicate. (C) Determination of TM of 2 independent experiments by sequence analysis of locus specific amplicons (454 Roche). On average, 10,000 amplicons were sequenced per experiment. Dark blue, insertion events; light blue, deletion events. The inset graph represents, as a function of insertion size, the percentage of meganuclease-induced TM with (green) or without (blue) Tdt. (D) Targeted mutagenesis at endogenous loci quantified by amplicon sequencing analysis. Meganucleases RAG1m, DMD21m (left panel) or CAPNS1m (right panel) were used either alone (empty) or with Tdt. Percentages of induced-TM are depicted as well the size of DNA insertions (inset graph). The mean percentage of insertion measured on the 3 endogenous loci is depicted.

To measure NHEJ activity induced by an engineered meganuclease (MN), a cellular model bearing a single copy of a transgene, depicted in Figure 1A, was developed in a 293H cell line. The transgene consists of a GFP open reading frame inactivated via a frame-shift introduced by cloning a 121 bp DNA sequence containing a meganuclease recognition site (5′ctgccccagggtgagaaagtccaa-3′) directly after the ATG start codon. Following DNA cleavage by the engineered meganuclease (GS, previously described [11]), inaccurate repair of a site-specific DSB by NHEJ could in principle restore the GFP reading-frame and thus indicate targeted disruption. Transfection of this cellular model with a meganuclease resulted in 0.6% GFP-positive cells as detected by flow cytometry 3 days post-transfection (Figure 1B). Molecular analysis of the entire cell population by amplicon sequencing revealed 3.2%±0.4 targeted mutagenesis (TM) events, of which 18% were TM events (or 0.6% of the total population) that restore the GFP coding frame, consistent with results obtained by flow cytometry.

DNA cleavage by meganucleases generates 3′-protruding single-strand ends that can be substrates for DNA-end processing enzymes such as polymerases or exonucleases. To determine if such enzymes could modify the frequency or type of repair events obtained in the presence of meganucleases, we first examined the impact of terminal deoxynucleotidyltransferase (Tdt) on TM. Tdt is a template-independent DNA polymerase that catalyzes the addition of deoxynucleotides to the 3′-hydroxyl terminus of oligonucleotide primers. It is expressed specifically in lymphoid cells during V(D)J recombination, increasing antigen receptor diversity by adding nucleotides at the coding ends of immunoglobulin and T cell receptor gene segments [32], [33], [34] Co-transfection of cells with Tdt and meganuclease leads to a 3-fold increase in GFP-positive cells (Figure 1B, compare 1.8% to 0.6% with the meganuclease alone). However, molecular analysis of the locus revealed a 8.2-±0.14 (p<0.0005) fold increase (26.9% vs. 3.2%) in the TM frequency in the Tdt co-transfected samples. This difference can be explained by the nature of the mutagenic events in the presence of Tdt, with 77% of all TM events being 2 to 3 base-pair insertions (Figure 1C) that in our cellular model do not restore a functional GFP gene.

To monitor the effect of Tdt on TM at endogenous loci, we used three site-specific engineered meganucleases, RAG1m, DMD21m and CAPNS1m, that target the human genes RAG1, DMD and CAPNS1, respectively (Data S1). In the absence of Tdt, meganuclease expression in human 293H cells results in TM frequencies of 1.5% to 18% depending on the locus (Figure 1D). In contrast, co-transfection with Tdt stimulated TM 2.5±0.33 fold (p<0.005), resulting in mutagenesis frequencies of up to 40%. Molecular analysis revealed a characteristic Tdt “signature” (i.e. small insertions events with no concomitant loss of nucleotides) represents the majority of the mutagenic events (mean 78%±5, p<0.0005) from 70 to 90% of TM) with no effect on the deletion pattern (Figure 1D, Figure S1, and Table S1). Tdt is known to interact with Ku via a BRCT-like sequence [35] and could impact the DNA-PK, XRCC4/NHEJ1/LIG4 dependent NHEJ (D-NHEJ) pathway. Thus, we can hypothesize that Tdt activity, by modifying the protruding DNA ends before being rejoined by D-NHEJ, “reveals” the large number of meganuclease cleavage events that are otherwise undetectable.

**Figure 2:**
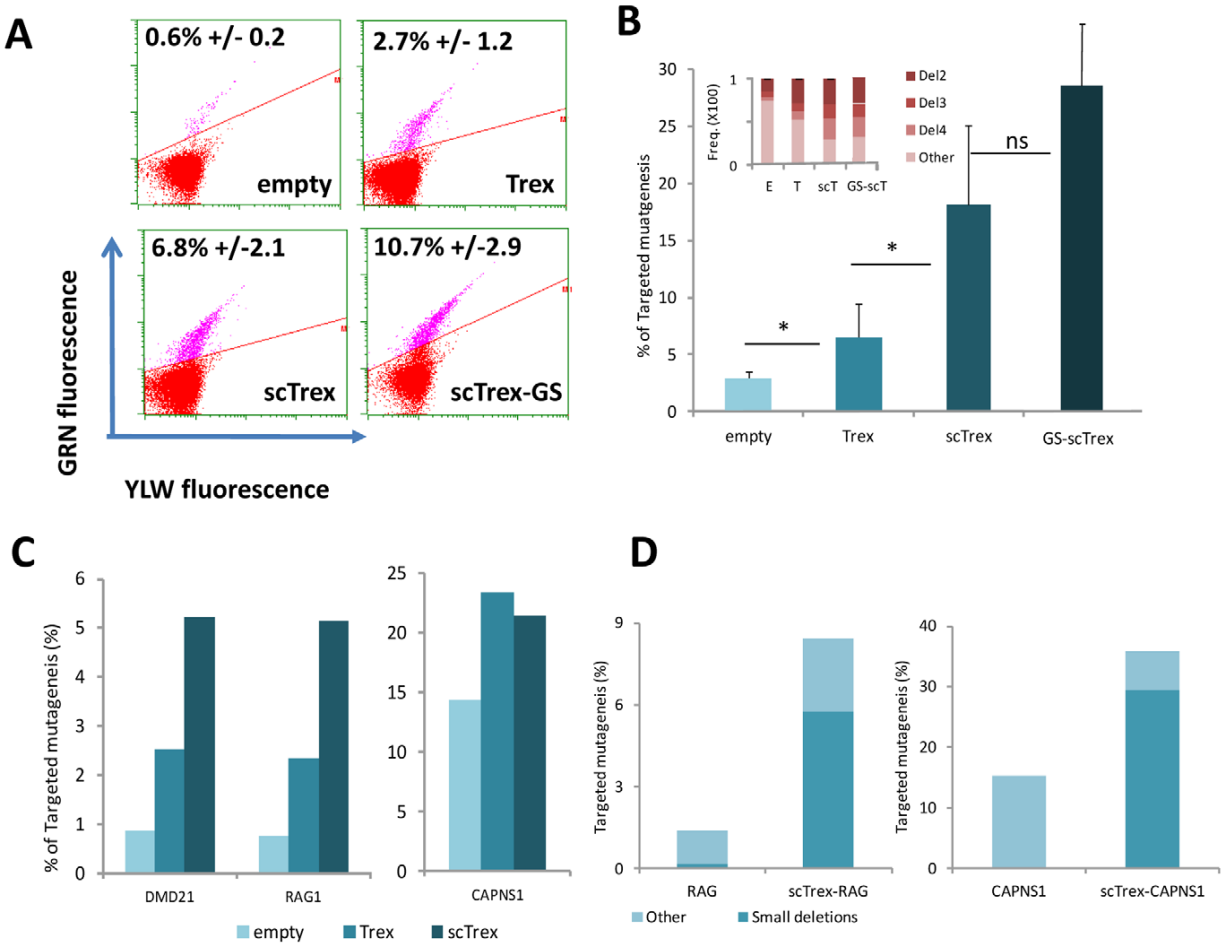
Effect of Trex2, scTrex or scTrex fused to meganuclease on meganuclease-induced mutagenesis. (A) Quantification by flow cytometry of the percentage of GFP positive cells 3 days post transfection with meganuclease alone (empty), with meganuclease and Trex2 or scTrex and meganuclease fused to scTrex; experiments performed in triplicate. (B) Determination of meganuclease-induced TM by sequence analysis of locus specific amplicons in the presence of wild-type Trex2 (Trex2), the engineered single-chain variant (scTrex) or meganuclease fused to scTrex. The inset graph shows the percentage of 2, 3, and 4 nucleotide (Del2, Del3, Del4) deletions among all TM events. E, meganuclease alone; T, meganuclease with Trex2; scT, meganuclease with scTrex. (C) Targeted mutagenesis at endogenous loci quantified by amplicon sequencing analysis. On average 10,000 amplicons were sequenced per experiment. Percentage of TM induced by meganucleases RAG1m, DMD21m (left panel) or CAPNS1m (right panel) are depicted. Empty, meganuclease alone; Trex2, meganuclease with wild-type Trex2. (D) scTrex was fused, respectively, to meganucleases targeting the hCAPNS1 and hRAG1 genes, and TM was determined by sequence analysis of locus specific amplicons.

As the preceding experiment demonstrated that TM was stimulated by addition of nucleotides to protruding DNA ends, it follows that nucleotide deletions would have a similar impact on meganuclease-induced mutagenesis. To this end, the human three prime repair exonuclease (TREX2) was selected for further study. Trex2 is a non-processive 3′ exonuclease [36] shown to degrade the 3′ DNA overhangs generated by the I-SceI meganuclease [37]. As Trex2 naturally functions as a homodimeric protein [38], we hypothesized that engineering a monomeric variant could enhance its exonucleolytic activity. Single-chain Trex2 (scTrex) was generated by fusing the C-terminus of one Trex2 monomer to the N-terminus of another via a flexible peptide linker (Data S2). The potential impact on TM by either wild-type Trex2 or the engineered scTrex variant was assayed using our GFP cellular model. In contrast to the 0.6% GFP positive cells induced by the meganuclease alone, addition of Trex2 or scTrex increased the frequency of GFP positive cells to 2.7% or 6.8%, respectively (Figure 2A). Molecular analysis of the locus by amplicon sequencing (454 Roche) confirmed this observation with increases in the 3.2% meganuclease-alone TM frequency to 6.4% and 18.1% in the presence of Trex2 and scTrex, respectively (Figure 2B). Induced mutagenic events are mainly small deletions of 2 to 4 nucleotides, consistent with degradation of the 3′ protruding DNA ends generated upon meganuclease cleavage. Trex2 exonuclease thus strongly enhances meganuclease-induced TM, with the monomeric single-chain design (scTrex) appearing more effective than the homodimeric wild-type.

The impact of Trex2 or scTrex on meganuclease-induced TM was subsequently investigated at three endogenous loci: RAG1, DMD21 and CAPNS1. Analysis of these loci upon appropriate meganuclease expression shows that TM frequencies of 0.8 to 14% can be achieved in human 293H cells in the absence of exogenous exonuclease activity (Figure 2C). As in the GFP model, scTrex is again more effective, stimulating the efficiency of TM up to 6-fold with resulting frequencies of ∼5% for the RAG1 and DMD loci and ∼23% for the CAPNS1 locus (Figure 2C). Detected events are associated with small deletions of 2 to 4 nucleotides, representing more than 60% of all TM events (Figure S2).

Although scTrex consistently outperformed the wild-type molecule in all our assays, we reasoned that we could further enhance its effectiveness by localizing the scTrex activity to the meganuclease cleavage site. To this end, scTrex-meganuclease fusions were generated by linking the C-terminus of scTrex to the N-terminus of three different meganucleases: GS, RAG1m and CAPNS1m (Data S2). Resulting chimeric meganucleases exhibited properties comparable to their respective parent proteins as evidenced by cleavage activity measured using an extrachromosomal SSA assay in CHO-K1 cells as well as toxicity profiles (Figure S3). The scTrex-GS fusion protein resulted in a significant increase in TM frequency in our GFP cellular model, with 10.7% events compared to 6.8% with co-transfection (Figure 2A). Molecular analysis shows that the meganuclease-alone induced frequency of 2.9%±0.6 TM can be boosted to 29.2%±9.2 using the chimeric construct (Figure 2B). Similar enhancements in meganuclease-induced events were observed for the scTrex-RAG1 and scTrex-CAPNS1 proteins tested on endogenous targets in 293H cells. Whereas expression of RAG1m or CAPNS1m alone leads to 1.4% or 15% TM events, respectively (Figure 2D), the chimeric scTrex-RAG1 and scTrex-CAPNS1 generate TM frequencies of 8.4% and 35.8%, respectively. As previously observed, the majority (70%) of TM events are small deletions of 2 to 4 nucleotides (Figure S4). Moreover, close examination of the TM induced by RAG1m revealed that 44% of the events correspond to a 9 nucleotide deletion resulting from a 5 bp micro-homology located within RAG1 target site. Additionally, although CAPNS1m induces large sequence deletions, the use of scTrex-CAPNS1 virtually eliminated these events (Figure S4).

**Figure 3:**
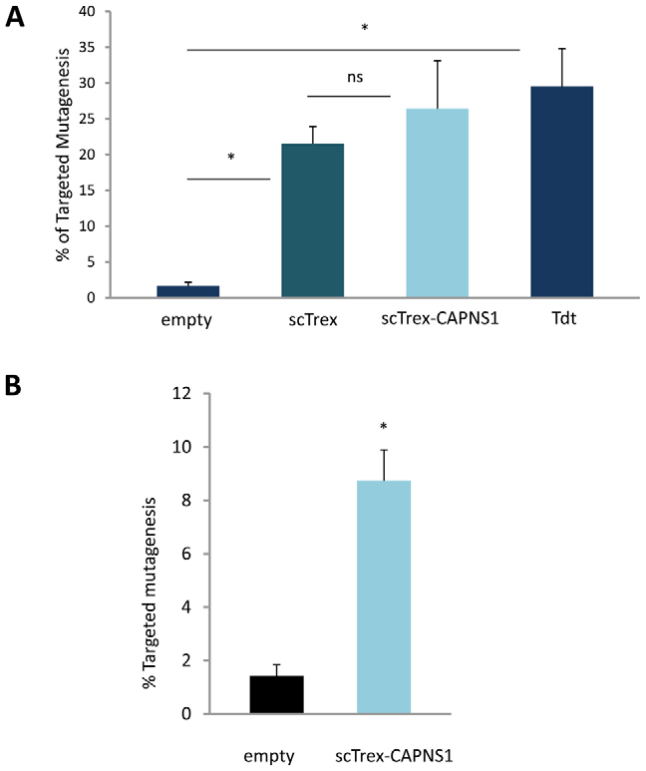
Targeted mutagenesis in primary cells and iPS. Cells were transfected with the CAPNS1m meganuclease in the absence (empty) or presence of DNA-end processing enzymes; scTrex, single-chain Trex2; scTrex-CAPNS1, meganuclease fused to scTrex; Tdt, terminal deoxynucleotidyltransferase. Frequency of TM as determined by sequence analysis of locus specific amplicons; ns, not significant. (A) Frequency of TM measured in Detroit 551 human fetal fibroblasts in 2 independent experiments. (B) Frequency of TM measured in iPS in 2 independent experiments.

Analogous results were obtained in experiments using Detroit 551 human fetal primary fibroblasts (D551). In this cell type, expression of CAPNS1m alone leads to 1.6%±0.5 TM events while co-expression of scTrex and scTrex-CAPNS1 or TdT increases the meganuclease-induced TM frequencies to 21.5%±2.4 and 26.4%±6.7 or 27.5%±2.5, respectively (Figure 3A). As in 293H cells, the respective Trex2 and TdT hallmarks of mutagenesis are observed in D551 cells, with nearly 90% small deletions (Trex2, Figure S5A) or insertions (Tdt, Figure S5B) among the TM events. Interestingly, CAPNS1m alone almost exclusively induced large deletions in 293H cell genome (Figure S2) yet it produced about 50% of small deletions within the D551 cell genome (Trex2, Figure S5A). Thus, despite differences in mutagenic events induced by the meganuclease in each cell type, the use of Trex2 or scTrex allows for better controlling TM outcomes in both cell types by restricting the majority of events to small deletions. Similar TM enhancement was obtained in human iPS cells (Figure 3B) in which expression of CAPNS1m alone leads to 1.4% ±0.4 TM events while expression of scTrex-CAPNS1 increases the meganuclease-induced TM frequencies to 8.7%±1.2.

Notably, although all meganucleases used in this study display similar intrinsic activities on extrachromosomal targets, we observed significant differences in their efficacy to induce gene modification at the endogenous locus (18% for CAPNS1m versus ∼1.5% for RAG1m and DMD21m). Such variations can be reconciled by considering chromatin compaction, given that the CAPNS1 target is more accessible to micrococcal enzyme than either the RAG1 or DMD21 targets in 293H cell lines. It is thus likely that impaired access to target, for both the meganuclease and proteins involved in DSB repair, may strongly influence the deletion/insertion patterns observed (Figures 2 and S2). Target-site accessibility could also explain the disparity in efficacy obtained for the same locus in different cell types. Indeed, CAPNS1m displays a lower activity in Detroit 551 cells than in 293H cells (1.6% versus 18%, respectively), leading to a TM profile similar to that obtained with RAG1m and DMD21m in 293H cells. Taken together, these observations suggest that depending on target location within the genome or the cell-type used in a particular experiment, nuclease-based technology may lead to various genome modification efficacies. Nevertheless, despite these heterogeneities, this study shows that the use of DNA processing enzymes increases meganuclease-induced TM and prevents the variation of mutagenic events due to the presence of micro–homologies, as shown for the RAG1 locus with enrichment in the 9-bp deletion event.

## Conclusions

Herein we describe a new strategy using DNA-end processing enzymes together with site-specific engineered endonucleases such as meganucleases to enable high rates of targeted mutagenesis. This approach is particularly suitable in cells with an unaltered D-NHEJ DNA maintenance pathway, wherein scarless rejoining of meganuclease-induced DSBs represents the majority of events [38] and thus genetic information is preserved. The efficiency of TM can be enhanced up to 30-fold using polymerases or exonucleases such as Tdt or Trex2 to add or remove nucleotides to or from the liberated DNA ends following cleavage. Our method allowed for achieving an exceptional 31% TM in primary cells when using meganuclease-induced mutagenesis. Tdt as well as Trex2 additionally offer the possibility to use low activity enzymes and to “control” the type of mutagenic events induced, for example, by favoring small insertions or deletions. While the use of Tdt leads to 70–90% small insertions (2 to 6 nucleotides), Trex2 usage results in 70–90% small deletions (2 to 4 nucleotides); both of which are independent of the locus or cell type. Finally, targeting exonuclease activity to the DSB via fusion to the specific endonuclease itself improves efficiency.

While it is conceivable that addition of DNA-end processing enzymes could be deleterious for genome integrity by increasing off-target mutagenic rates, we did not observe any toxicity either in survival assays or growth rate analyses for cell populations transfected with meganucleases and/or DNA-end processing enzymes. However, as noted above, target-site accessibility is a crucial parameter in the DSB repair process that could as well affect the potential off-target mutagenesis frequency. Moreover, beyond modulating the nature of the mutagenic events, Trex2 over-expression may be beneficial for nuclease-based genome modification. Recently, Bennardo *et al.* published data suggesting that Trex2 over-expression decreases the frequency of non-homologous end-joining between distal ends of two DSBs by restricting the persistence of target sites available for repeated cleavage-ligation cycles. In principle this limits the persistence of off-target DNA breaks and thus reduces potential deleterious translocations. Altogether, our study shows that coupling DNA end-processing enzymes with meganucleases enhances targeted mutagenesis and represents an attractive new tool for precise genome engineering purposes.

## Supporting Information

Data S1 **Meganucleases sequences.** Monomers A and B are linked by the linker GGSDKYNQALSKYNQALSKYNQALSGGGGS. 19S mutation is added in monomer B.
(DOC)

Data S2 **Amino acids sequences of the different hybrid proteins used in this study.** scTrex2: Single chain molecule of Trex2 exonuclease. Two Trex2 monomers were fused together using the linker 1 : TPPQTGLDVPY. ScTrex-meganuclease: meganuclease results from the fusion of two engineered monomers linked by linker 2.. The single chain meganuclease was then fused by its N-t domain to the single chain Trex2 using the linker 3. The resulting molecule harbors endonuclease and 3′->5′exonuclease activity.
(DOCX)

Figure S1 **Deletion pattern induced by MN in the presence of Tdt.** Deletion events induced by RAG1m (A) DMD21m (B) or CAPNS1m (C) in the presence (green) or absence (blue) of Tdt were analyzed. Deletion sizes are represented as percentage of total deletion events. Note that CAPNS1m induces large and diverse deletions that were analyzed by deletion classes (range of deletions size).
(EPS)

Figure S2 **Targeted mutagenesis pattern induced by MNs in the presence of Trex or scTrex.** Percentages of TM events induced by RAG1m (A) DMD21m (B) or CAPNS1m (C) with or without (empty) Trex or scTrex are shown. Del2, 2 bp deletion; Del3, 3 bp deletion; Del4, 4 bp deletion; Other, more than 4 bp Deletions as well as other TM events induced by RAG1m (a) DMD21m (b) or CAPNS1m (c) with or without (empty) Trex or scTrex.
(EPS)

Figure S3 **MNs activity and toxicity in the presence of Trex or scTrex.** (A) *In vivo* cleavage activity of MNs alone or fused to scTrex. Activity was monitored using an extrachromasomal SSA assay. CHO-K1 cells were transfected with increasing amounts (0 to 25 ng) of MN or chimeric scTrex-MN and tested against their respective targets. Empty vector and I-SceI are shown as controls. (B) Toxicity of the chimeric scTrex-MN proteins was evaluated in a cell survival assay. CHO-K1 cells were co-transfected with increasing amounts of MN or scTrex-MN in the presence of 10 ng of plasmid expressing GFP. Cell survival is expressed as a percentage of cells expressing GFP 6 days post transfection ^23^.
(EPS)

Figure S4 **Deletion pattern induced by MNs and scTrex-MNs.** Deletion events induced at the RAG1 (A) or CAPNS1 (B) locus by MN alone (blue) or scTrex-MN (green) are shown. Deletion sizes are represented as percentage of total deletion events. Note that 45% of TM induced by RAG1m corresponds to a 9 bp deletion whereas CAPNS1m induces large and diverse deletions that were analyzed by deletion classes (range of deletions size).
(EPS)

Figure S5 **Targeted mutagenesis pattern in Detroit 551 cells induced by CAPNS1m in the absence (empty) or presence of scTrex, single-chain Trex2; scTrex-CAPNS1, MN fused to scTrex; Tdt, terminal deoxynucleotidyltransferase.** (A) Frequency of small deletion events (2 to 4 nucleotides) among all TM events. (B) Profile of insertion events in the presence (green) or absence (blue) of Tdt.
(EPS)

Table S1 **Examples of sequences with insertion at transgenic locus in presence of Tdt.** Sequences marked with asterisk (*) correspond to insertion events independent of the TDT activity. They are also found in the sample corresponding to cells transfected with meganuclease in absence of TDT.
(DOC)

Table S2 **Targeted mutagenesis data. Nucleases encoding plasmids were transfected with or without DNA-end processing enzyme encoding plasmids in 10 µg of total DNA.** Cells were harvested 3 days post-transfection for genomic DNA extraction and locus specific PCR amplification for deep sequencing analysis. Several thousands of sequences were obtained per PCR product and then analyzed for site-specific insertion or deletion events.
(DOC)
