# Supplementary material for: High Frequency Targeted Mutagenesis Using Engineered Endonucleases and DNA-End Processing Enzymes
Source: PLoS One. 2013 Jan 24;8(1):e53217. doi: 10.1371/journal.pone.0053217 (PMC3554739; doi:10.1371/journal.pone.0053217)
Supplement: Data S1 — Meganucleases sequences. Monomers A and B are linked by the linker GGSDKYNQALSKYNQALSKYNQALSGGGGS. 19S mutation is added in monomer B. (DOC) [file pone.0053217.s001.doc]

**Supporting data 1**

| Meganucleases | Mutations potentially involved in DNA recognition  Monomer A _ Monomer B |
| --- | --- |
| GSm | 30R38E44D68Y70S75Y_30R68A |
| CAPNS1m | 24V68Q70S75N_30R44Y68E70S75R |
| RAG1m | 38R44Y70S75Q_30R44A68Y70S |
| DMD21m | 30R44T46G68T70S73M75A_38Y70S |

I-CreIm

MANTKYNKEFLLYLAGFVDGDGSIIAQIKPNQSYKFKHQLSLTFQVTQKTQRRWFLDKLVDEIGVGYVRDRGSVSDYILSEIKPLHNFLTQLQPFLKLKQKQANLVLKIIEQLPSAKESPDKFLEVCTWVDQIAALNDSKTRKTTSETVRAVLDSLSEKKKSSPAAD
