# Supplementary material for: High Frequency Targeted Mutagenesis Using Engineered Endonucleases and DNA-End Processing Enzymes
Source: PLoS One. 2013 Jan 24;8(1):e53217. doi: 10.1371/journal.pone.0053217 (PMC3554739; doi:10.1371/journal.pone.0053217)
Supplement: Data S2 — Amino acids sequences of the different hybrid proteins used in this study. scTrex2: Single chain molecule of Trex2 exonuclease. Two Trex2 monomers were fused together using the linker 1 : TPPQTGLDVPY. ScTrex-meganuclease: meganuclease results from the fusion of two engineered monomers linked by linker 2.. The single chain meganuclease was then fused by its N-t domain to the single chain Trex2 using the linker 3. The resulting molecule harbors endonuclease and 3′->5′exonuclease activity. (DOCX) [file pone.0053217.s002.docx]

**Supplementary data 2.**

scTrex2 :

MGSEAPRAETFVFLDLEATGLPSVEPEIAELSLFAVHRSSLENPEHDESGALVLPRVLDKLTLCMCPERP

FTAKASEITGLSSEGLARCRKAGFDGAVVRTLQAFLSRQAGPICLVAHNGFDYDFPLLCAELRRLGARLP

RDTVCLDTLPALRGLDRAHSHGTRARGRQGYSLGSLFHRYFRAEPSAAHSAEGDVHTLLLIFLHRAAELL

AWADEQARGWAHIEPMYLPPDDPSLEATPPQTGLDVPYSEAPRAETFVFLDLEATGLPSVEPEIAELSLF

Linker 1

AVHRSSLENPEHDESGALVLPRVLDKLTLCMCPERPFTAKASEITGLSSEGLARCRKAGFDGAVVRTLQA

FLSRQAGPICLVAHNGFDYDFPLLCAELRRLGARLPRDTVCLDTLPALRGLDRAHSHGTRARGRQGYSLG

SLFHRYFRAEPSAAHSAEGDVHTLLLIFLHRAAELLAWADEQARGWAHIEPMYLPPDDPSLEA

scTrex-meganuclease :

scTrex-CAPNS :

Engineered meganucleases described in this manuscript have all been described elwhere (Daboussi

MGSEAPRAETFVFLDLEATGLPSVEPEIAELSLFAVHRSSLENPEHDESGALVLPRVLDKLTLCMCPERP

FTAKASEITGLSSEGLARCRKAGFDGAVVRTLQAFLSRQAGPICLVAHNGFDYDFPLLCAELRRLGARLP

RDTVCLDTLPALRGLDRAHSHGTRARGRQGYSLGSLFHRYFRAEPSAAHSAEGDVHTLLLIFLHRAAELL

AWADEQARGWAHIEPMYLPPDDPSLEATPPQTGLDVPYSEAPRAETFVFLDLEATGLPSVEPEIAELSLF

Linker 1

AVHRSSLENPEHDESGALVLPRVLDKLTLCMCPERPFTAKASEITGLSSEGLARCRKAGFDGAVVRTLQA

FLSRQAGPICLVAHNGFDYDFPLLCAELRRLGARLPRDTVCLDTLPALRGLDRAHSHGTRARGRQGYSLG

SLFHRYFRAEPSAAHSAEGDVHTLLLIFLHRAAELLAWADEQARGWAHIEPMYLPPDDPSLEAGGGGSGG

Linker 3

GGSNTKYNEEFLLYLAGFVDGDGSIVAQIKPNQRAKFKHQLSLTFQVTQKTQRRWLLDKLVDEIGVGYVQ

DSGSVSNYRLSEIKPLHNFLTQLQPFLELKQKQANLVLKIIEQLPSAKESPDKFLEVCTWADQIAALNDS

KTRKTTSETVRAVLDSLSEKKKSSPAAGGSDKYNQALSKYNQALSKYNQALSGGGGSNKKFLLYLAGFVD

Linker 2

SDGSIIAQIKPRQSYKFKHQLRLTFYVTQKTQRRWFLDKLVDRIGVGYVEDSGSVSRYVLSEIKPLHNFL

TQLQPFLKLKQKQANLVLKIIEQLPSAKESPDKFLEVCTWVDQVAALNDSKTRKTTSETVRAVLDSLSEK

KKSSP

scTrex-RAG

MGSEAPRAETFVFLDLEATGLPSVEPEIAELSLFAVHRSSLENPEHDESGALVLPRVLDKLTLCMCPERP

FTAKASEITGLSSEGLARCRKAGFDGAVVRTLQAFLSRQAGPICLVAHNGFDYDFPLLCAELRRLGARLP

RDTVCLDTLPALRGLDRAHSHGTRARGRQGYSLGSLFHRYFRAEPSAAHSAEGDVHTLLLIFLHRAAELL

AWADEQARGWAHIEPMYLPPDDPSLEATPPQTGLDVPYSEAPRAETFVFLDLEATGLPSVEPEIAELSLF

Linker 1

AVHRSSLENPEHDESGALVLPRVLDKLTLCMCPERPFTAKASEITGLSSEGLARCRKAGFDGAVVRTLQA

FLSRQAGPICLVAHNGFDYDFPLLCAELRRLGARLPRDTVCLDTLPALRGLDRAHSHGTRARGRQGYSLG

SLFHRYFRAEPSAAHSAEGDVHTLLLIFLHRAAELLAWADEQARGWAHIEPMYLPPDDPSLEAGGGGSGG

Linker 3

GGSNTKYNEEFLLYLAGFVDGDGSIIAQINPNQSSKFKHRLRLTFYVTQKTQRRWFLDKLVDEIGVGYVR

DSGSVSQYVLSEIKPLHNFLTQLQPFLELKQKQANLVLKIIEQLPSAKESPDKFLEVCTWVDQIAALNDS

KTRKTTSETVRAVLDSLSGKKKSSPAAGGSDKYNQALSKYNQALSKYNQALSGGGGSNKKFLLYLAGFVD

Linker 2

SDGSIIAQIKPRQSNKFKHQLSLTFAVTQKTQRRWFLDKLVDRIGVGYVYDSGSVSDYRLSEIKPLHNFL

TQLQPFLKLKQKQANLVLKIIEQLPSAKESPDKFLEVCTWVDQIAALNDSKTRKTTSETVRAVLDSLSEK

KKSSP

scTrex-GS

MGSEAPRAETFVFLDLEATGLPSVEPEIAELSLFAVHRSSLENPEHDESGALVLPRVLDKLTLCMCPERP

FTAKASEITGLSSEGLARCRKAGFDGAVVRTLQAFLSRQAGPICLVAHNGFDYDFPLLCAELRRLGARLP

RDTVCLDTLPALRGLDRAHSHGTRARGRQGYSLGSLFHRYFRAEPSAAHSAEGDVHTLLLIFLHRAAELL

AWADEQARGWAHIEPMYLPPDDPSLEATPPQTGLDVPYSEAPRAETFVFLDLEATGLPSVEPEIAELSLF

Linker 1

AVHRSSLENPEHDESGALVLPRVLDKLTLCMCPERPFTAKASEITGLSSEGLARCRKAGFDGAVVRTLQA

FLSRQAGPICLVAHNGFDYDFPLLCAELRRLGARLPRDTVCLDTLPALRGLDRAHSHGTRARGRQGYSLG

SLFHRYFRAEPSAAHSAEGDVHTLLLIFLHRAAELLAWADEQARGWAHIEPMYLPPDDPSLEAGGGGSGG

Linker 3

GGSNTKYNEEFLLYLAGFVDADGSIIAQIKPRQSRKFKHELSLTFDVTQKTQRRWFLDKLVDEIGVGYVY

DSGSVSYYQLSEIKPLHNFLTQLQPFLELKQKQANLVLKIIEQLPSAKESPAKFLEVCTWVDQIAALNDS

KTRKTTSETVRAVLDSLSEKKKSSPAAGGSDKYNQALSKYNQALSKYNQALSGGGGSNKKFLLYLAGFVD

Linker 2

GDGSIIAQIKPRQGYKFKHQLSLTFQVTQKTQRRWFLDKLVDRIGVGYVADRGSVSDYRLSEIKPLHNFL

TQLQPFLKLKQKQANLVLKIIEQLPSAKESLDKFLEVCTWVDQIAALNDSKTRKTTSETVRAVLDSLSEK

KKSSP
