# Supplementary material for: High Frequency Targeted Mutagenesis Using Engineered Endonucleases and DNA-End Processing Enzymes
Source: PLoS One. 2013 Jan 24;8(1):e53217. doi: 10.1371/journal.pone.0053217 (PMC3554739; doi:10.1371/journal.pone.0053217)
Supplement: Table S1 — Examples of sequences with insertion at transgenic locus in presence of Tdt. Sequences marked with asterisk (*) correspond to insertion events independent of the TDT activity. They are also found in the sample corresponding to cells transfected with meganuclease in absence of TDT. (DOC) [file pone.0053217.s008.doc]

Table S1

A: example of sequences with insertion at transgenic locus in presence of Tdt.

| Sequences with insertion | Insertion size |
| --- | --- |
| ctgccccagggtgaaagaaagtccaa | 2 |
| ctgccccagggtgagggaaagtccaa | 2 |
| ctgccccagggtgaacgaaagtccaa | 2 |
| ctgccccagggtgagagaaagtccaa | 2 |
| ctgccccagggtgtgagaaagtccaa | 2 |
| ctgccccagggtgatagaaagtccaa | 2 |
| ctgccccaggccgtgagaaagtccaa | 2 |
| ctgccccaggtcgtgagaaagtccaa | 2 |
| ctgccccagggtgaagagaaagtccaa | 3 |
| ctgccccagggtgacgggaaagtccaa | 3 |
| ctgccccagggtcgtgagaaagtccaa | 3 |
| ctgccccaggttcgtgagaaagtccaa | 3 |
| ctgccccagggtgaagggaaagtccaa | 3 |
| ctgccccagggcccgtgagaaagtccaa | 4 |
| ctgccccaggccttgtgagaaagtccaa | 4 |
| ctgccccagggtgaaggagaaagtccaa | 4 |
| ctgccccagggtgatagagaaagtccaa | 4 |
| ctgccccagggtgaagcgggaaagtccaa | 5 |
| *ctgccccagggtgaagtgagaaagtccaa | 5 |
| ctgccccagggccccgtgagaaagtccaa | 5 |
| *ctgccccagggtgaaggtgagaaagtccaa | 6 |
| ctgccccaggggcggagtgagaaagtccaa | 6 |

B: example of sequences with insertion at RAG1 locus in presence of Tdt.

| Sequences with insertion | Insertion size |
| --- | --- |
| attgttctcaggcgtacctcagccagc | 2 |
| attgttctcaggtacatctcagccagc | 2 |
| attgttctcaggtacccctcagccagc | 2 |
| attgttctcaggtacgggctcagccagc | 3 |
| attgttctcagggcgtacctcagccagc | 3 |
| attgttctcaggtacagtctcagccagc | 3 |
| attgttctcaggtacggggctcagccag | 4 |
| attgttctcagacccgtacctcagccagc | 4 |
| attgttctcagcctcgtacctcagccagc | 4 |
| attgttctcagcttcgtacctcagccagc | 4 |
| attgttctcaggtactggactcagccagc | 4 |
| attgttctcaggtacagggctcagccagc | 4 |
| attgttctcaggtactgacctcagccagc | 4 |
| attgttctcaggtacgggaactcagccagc | 5 |
| attgttctcaggtacgaaggctcagccagc | 5 |
| attgttctcagttcctgtacctcagccagc | 5 |
| attgttctcaggtacgggtggctcagccagc | 6 |
| attgttctcaggtactggttactcagccagc | 6 |
| attgttctcaggtacccatacctcagccagc | 6 |
| attgttctcaggttacctgtacctcagccagc | 7 |
| attgttctcaggtacaagggggctcagccagc | 7 |
| attgttctcagggccgcccgtacctcagccagc | 8 |

Note that WT sequence of Rag1 present 5bp homologies (unbold): attgtt*ctcag*gtac*ctcag*ccagc

C: example of sequences with insertion at CAPNS1 locus in presence of Tdt.

| Sequences with insertion | Insertion size |
| --- | --- |
| cagggccgcggtgcgcagtgtccgac | 2 |
| cagggccgcgccgtgcagtgtccgac | 2 |
| cagggccgcggcgtgcagtgtccgac | 2 |
| cagggccgcggtgcacagtgtccgac | 2 |
| cagggccgcggtgcgaagtgtccgac | 2 |
| cagggccgcggtgcccagtgtccgac | 2 |
| cagggccgcggtgcggagtgtccgac | 2 |
| cagggccgcggccgtgcagtgtccgac | 3 |
| cagggccgcggtgctgcagtgtccgac | 3 |
| cagggccgcgcctgtgcagtgtccgac | 3 |
| cagggccgcgttctgtgcagtgtccgac | 4 |
| cagggccgcggtgcgggcagtgtccgac | 4 |
| cagggccgcggtccgtgcagtgtccgac | 4 |
| cagggccgcggtgcaggcagtgtccgac | 4 |
| cagggccgcggtgcaaagcagtgtccgac | 5 |
| *cagggccgcggtgcagtgcagtgtccgac | 5 |
| cagggccgcggtgcggtgcagtgtccgac | 5 |
| cagggccgcgtgtctgtgcagtgtccgac | 6 |
| cagggccgcggtgcaaggtcagtgtccgac | 6 |
| *cagggccgcggtgcccgtgcagtgtccgac | 6 |
| *cagggccgcggtgcaagtgcagtgtccgac | 6 |
| cagggccgcggtgcaagcagggagtgtccgac | 8 |

D: example of sequences with insertion at DMD21 locus in presence of Tdt.

| Sequences with insertion | Insertion size |
| --- | --- |
| tttacatttggtactcttgaggttt | 2 |
| tttacatttgtggtacttgaggttt | 2 |
| tttacatttgcggtacttgaggttt | 2 |
| tttacatttggtacacttgaggttt | 2 |
| tttacatttggtacgacttgaggttt | 3 |
| tttacatttggtacgggttgaggttt | 3 |
| tttacatttgccggtacttgaggttt | 3 |
| tttacatttggtacgaattgaggttt | 3 |
| tttacatttcccggtacttgaggttt | 3 |
| tttacatttggtacaggcttgaggttt | 4 |
| tttacatttccctggtacttgaggttt | 4 |
| tttacatttggtacgaccttgaggtt | 4 |
| tttacatttggtacgaagttgaggttt | 4 |
| tttacatttcctcggtacttgaggttt | 4 |
| tttacatttgtcaggtacttgaggttt | 4 |
| tttacatttcctcggtacttgaggttt | 4 |
| tttacatttggtccggtacttgaggttt | 5 |
| tttacatttggtacagctcttgaggttt | 5 |
| tttacatttccccctggtacttgaggttt | 6 |
| *tttacatttggtacgggtacttgaggttt | 6 |
| *tttacatttggtacccggtacttgaggttt | 7 |
| *tttacatttggtacagaggacttgaggttt | 7 |
