# Supplementary material for: High Frequency Targeted Mutagenesis Using Engineered Endonucleases and DNA-End Processing Enzymes
Source: PLoS One. 2013 Jan 24;8(1):e53217. doi: 10.1371/journal.pone.0053217 (PMC3554739; doi:10.1371/journal.pone.0053217)
Supplement: Table S2 — Targeted mutagenesis data. Nucleases encoding plasmids were transfected with or without DNA-end processing enzyme encoding plasmids in 10 µg of total DNA. Cells were harvested 3 days post-transfection for genomic DNA extraction and locus specific PCR amplification for deep sequencing analysis. Several thousands of sequences were obtained per PCR product and then analyzed for site-specific insertion or deletion events. (DOC) [file pone.0053217.s009.doc]

| **Figure** | **SubFigure** | **Sample** | **Sample size**  **(number of seq. analyezd)** | **Number of sequences with TM events** | **Insertions** | **Deletions** |
| --- | --- | --- | --- | --- | --- | --- |
| ***Figure 1c*** |  | empty | 3834 | 123 | 36 | 91 |
|  |  | Tdt | 5084 | 1324 | 1262 | 69 |
|  |  | empty | 7577 | 253 | 68 | 189 |
|  |  | Tdt | 20072 | 5575 | 5213 | 383 |
|  |  |  |  |  |  |  |
| ***Figure 1d*** | **CAPNS1** | empty | 945 | 172 | 16 | 158 |
|  |  | Tdt | 837 | 332 | 259 | 75 |
|  |  |  |  |  |  |  |
|  | **RAG1** | empty | 6291 | 109 | 3 | 106 |
|  |  | Tdt | 7273 | 360 | 264 | 98 |
|  |  |  |  |  |  |  |
|  | **DMD** | empty | 17823 | 225 | 29 | 202 |
|  |  | Tdt | 12486 | 389 | 326 | 66 |
|  |  |  |  |  |  |  |
| ***Figure 2b*** |  | empty | 7578 | 253 | 68 | 189 |
|  |  | empty | 32375 | 801 | 184 | 637 |
|  |  | Trex | 8868 | 755 | 81 | 680 |
|  |  | Trex | 30085 | 1276 | 145 | 1146 |
|  |  | scTrex | 9726 | 2243 | 46 | 2217 |
|  |  | scTrex | 23277 | 3092 | 58 | 3052 |
|  |  | scTrex-GS | 37909 | 8574 | 71 | 8546 |
|  |  | scTrex-GS | 16325 | 5828 | 66 | 5803 |
|  |  |  |  |  |  |  |
| ***Figure 2c*** | **CAPNS1** | empty | 6808 | 976 | 68 | 910 |
|  |  | Trex | 10230 | 2398 | 92 | 2312 |
|  |  | scTrex | 3692 | 791 | 70 | 725 |
|  |  |  |  |  |  |  |
|  | **DMD** | empty | 9066 | 78 | 27 | 60 |
|  |  | Trex | 12057 | 306 | 12 | 300 |
|  |  | scTrex | 7976 | 416 | 66 | 394 |
|  |  |  |  |  |  |  |
|  | **RAG** | empty | 11321 | 86 | 4 | 83 |
|  |  | Trex | 15046 | 368 | 22 | 349 |
|  |  | scTrex | 16202 | 833 | 27 | 811 |
|  |  |  |  |  |  |  |
| ***Figure 2d*** | **CAPNS1** | CAPNS1 | 6657 | 1014 | 86 | 936 |
|  |  | scTREX-CAPNS1 | 5832 | 2089 | 14 | 2077 |
|  |  |  |  |  |  |  |
|  | **RAG** | RAG | 1289 | 18 | 0 | 18 |
|  |  | scTREX-RAG | 902 | 76 | 0 | 76 |
|  |  |  |  |  |  |  |
| ***Figure 3*** | **Detroit** | empty | 3152 | 40 | 8 | 32 |
|  |  | empty | 4166 | 84 | 14 | 70 |
|  |  | scTrex | 3782 | 751 | 9 | 742 |
|  |  | scTrex | 525 | 122 | 6 | 117 |
|  |  | scTrex-CAPNS1 | 2746 | 856 | 2 | 855 |
|  |  | scTrex-CAPNS1 | 235 | 51 | 0 | 51 |
|  |  | Tdt | 3631 | 935 | 906 | 29 |
|  |  | Tdt | 3003 | 879 | 832 | 47 |
|  |  |  |  |  |  |  |
|  | **iPS** | empty | 7875 | 89 | 1 | 88 |
|  |  | empty | 8052 | 141 | 9 | 132 |
|  |  | scTrex-CAPNS1 | 2045 | 162 | 1 | 161 |
|  |  | scTrex-CAPNS1 | 1329 | 127 | 0 | 127 |
|  |  |  |  |  |  |  |

Table S2  : Targeted mutagenesis data.
